# Distinct ERP profiles for auditory processing in infants at-risk for autism and language impairment

**DOI:** 10.1038/s41598-017-19009-y

**Published:** 2018-01-15

**Authors:** Valentina Riva, Chiara Cantiani, Giulia Mornati, Martina Gallo, Laura Villa, Elisa Mani, Irene Saviozzi, Cecilia Marino, Massimo Molteni

**Affiliations:** 1 Child Psychopathology Unit, Scientific Institute, IRCCS Eugenio Medea, Bosisio Parini, Lecco Italy; 2 Centre for Addiction and Mental Health (CAMH) University of Toronto (Canada), Toronto, Canada

## Abstract

Early identification of autism spectrum disorder (ASD) is crucial for the formulation of effective intervention programs. Language deficits may be a hallmark feature of ASD and language delay observed in ASD shows striking similarities to that observed in children with language impairment (LI). Auditory processing deficits are seen in both LI and ASD, however, they have not previously been compared directly using Event-Related Potentials (ERPs) in the two at-risk populations. This study aims to characterize infants at-risk for ASD (HR-ASD) at the electrophysiological level and to compare them with infants at-risk for LI (HR-LI) and controls, to find specific markers with predictive value. At 12-month-old, auditory processing in HR-ASD, HR-LI and controls was characterized via ERP oddball paradigm. All infants were then evaluated at 20 months, to investigate the associations between auditory processing and language/ASD-related outcomes. In both HR-ASD and HR-LI, mismatch response latency was delayed compared to controls, whereas only HR-ASD showed overall larger P3 amplitude compared to controls. Interestingly, these ERP measures correlated with later expressive vocabulary and M-CHAT critical items in the whole sample. These results may support the use of objective measurement of auditory processing to delineate pathophysiological mechanisms in ASD, as compared to LI.

## Introduction

Autism Spectrum Disorder (ASD) is typically a lifelong neurodevelopmental condition that involves difficulties in communication and social interaction, and restricted, repetitive behavior and interests^1^. There is a general consensus that ASD is a complex heritable condition typically diagnosed early in life, with meta-analytic heritability estimates ranging between 64% and 91%^2^. However, behavioral symptoms do not emerge until 24 months of age, and a clinical diagnosis is often not received before the third birthday^3^. To help detect abnormalities arising before the behavioral symptoms and to identify early markers of the disorder, the research has turned towards studying infant siblings of children with ASD, referred to as at-high risk for developing ASD. In the Baby Siblings Research Consortium, it was found that 18.7% of 664 high-risk siblings developed an ASD at three years old^4^, and a recurrence of 19.5% in a larger at-high risk sample (*n = *1241) was successively replicated^5^.

Both language delays and atypical responses to sensory information seem to be more prevalent in the ASD population than other developmental disabilities^6^. Especially in the first years of life, deficits in language skills are a hallmark feature of ASD. Children with ASD experience difficulties in extracting the linguistic information received through auditory perception, and have been reported to speak their first words^7,8^ and first phrases^9,10^ later.

At the clinical level, delays in early language development are often the primary concern motivating parents to seek diagnostic evaluation of their children^11^. At the same time, deficits in sensory processing have been reported in up to 87% of patients^12–14^ and hyper- or hypo-reactivity to sensory input is now included in the new DSM-5, becoming part of the diagnostic criteria for ASD^1^. More importantly, severity of sensory features in children with ASD has been found to predict social-communication difficulties^15,16^.

As sensory processing is crucial for clinical diagnosis, and language deficits may be a core characteristic of ASD, studies of the role played by auditory processing functioning in early phases of life is crucial to examining the roots of ASD.

Overall, many researchers have reported abnormalities in low-level auditory processing in ASD, even if results are still mixed. Enhanced pitch perception, especially for simple auditory stimuli, has been reported in individuals with ASD^17^. The behavioral findings for enhanced frequency discrimination are supported by Event-Related Potential (ERP) evidence in children and adults with ASD. In particular, larger amplitudes and/or earlier latencies of selected ERP components have been found in response to pitch changes for simple auditory material^18–20^.

In contrast, different results have been obtained in the auditory domain when more spectro-temporally complex stimuli or paradigms have been used. Converging ERP and neuroimaging studies reported that individuals with ASD show impaired auditory processing skills in tasks involving spectrally and/or temporally complex stimuli. Some authors suggested that the advantage shown by ASD individuals when processing basic auditory stimuli such as tones or consonant-vowel syllables was lost when the context of the stimuli was meaningful (e.g. words) and required abstracting invariant speech features from varying input^21–23^. Thus, auditory processing impairments in ASD seem to be more related to auditory temporal-envelope processing with a high temporal and spectral demand, rather than to simple pitch information processing^17,24^.

Against this background, it should not be surprising that extensive research has proposed auditory processing deficits as the underlying cause of language impairment - LI^25^. According to the temporal processing deficit hypothesis, individuals with LI have problems in processing auditory elements characterized by rapid transitions and short durations^25–27^. Given that most speech sounds involve brief, rapidly succeeding intra-syllabic acoustic changes, auditory processing deficits compromise the temporal analysis of speech at the phonemic level, and thus the building of correct phoneme representations. Additional research, using both behavioral and ERP techniques, has shown that school-aged children with LI exhibit deficits for both speech and non-speech sound processing, including detecting amplitude and frequency modulations^28^, as well as changes in sound frequency, duration, and onset rise time. These anomalies are already detectable in infants at-high risk for LI^29–31^ and are predictive of preschool expressive and receptive language outcomes^29,32,33^.

Interestingly, language impairment observed in the two clinical populations (ASD and LI) shows striking similarity, especially in the first years of life. In addition, genetic studies have shown familial clustering with ASD and LI, further suggesting that there may be some overlapping genetic etiology between the two disorders^34^.

Taken together, while many typical ASD symptoms can differentiate from LI, previous findings converge to suggest that both individuals with ASD and LI demonstrate behavioral and neurophysiological abnormalities in response to auditory processing at both early and later stages of processing. However, the two disorders have not been directly compared during the early phases of life and there is no ERP study on auditory processing comparing infants at-high risk for ASD and LI.

For the first time, we tested infants at 12 months and we measured electrophysiological responses to auditory processing in two at-risk groups (20 infants at-high risk for ASD - HR-ASD, and 19 infants at-high risk for LI - HR-LI) compared with 22 typically developing infants, to find specific electrophysiological markers with predictive and prognostic value. Furthermore, all infants were then evaluated at 20 months of age, to investigate the association between early auditory processing and ASD-related traits and language skills.

The use of functional brain measures such as EEG and ERP techniques offers more insight, as these measures uncover infants’ perception and processing abilities without being restricted to infants’ behavioral capabilities. Previous research has proposed specific ERP components as neural underpinnings of the change detection process in infants. Specifically, in “oddball” paradigms (in which ‘deviant’ stimuli are occasionally and randomly introduced into a sequence of more frequent ‘standard’ stimuli), a large positivity at about 300 ms from ‘deviant’ stimulus onset has been reported^35^. This component is often referred to as the P3 component—so named to reflect its polarity and average time of onset. In the difference ERP waveform (obtained by calculating the difference between responses evoked by ‘deviant’ versus ‘standard’ stimuli), the resulting component is labeled the mismatch response (MMR) in infant data^36^. Both these components (P3 and MMR) have been reported as possible markers of the neural change detection process underlying vulnerability to both language and ASD-related impairments^17,29,30^. In the present study, a multi-feature oddball paradigm was introduced to elicit P3/MMR responses for rapidly-presented pairs of complex tones (‘standard’ stimuli, STD) and for two types of ‘deviant’ tone-pairs, with differences in fundamental frequency (DEVF) and variation in sound duration (DEVD). This specific paradigm was successfully used in previous works^29,37,38^ in order to elicit comparable responses for the two auditory attributes of interest within the same paradigm in the same sample of infants.

The aim of the study was twofold: (1) to explore whether and to what extent P3/MMR measures could be used to compare and differentiate HR groups from controls and (2) to identify the association between early auditory processing and later development by assessing correlations between P3/MMR components at 12 months and language and ASD-related traits at 20 months.

Based on the literature suggesting hyper-sensitivity to low-level auditory stimuli in ASD, we hypothesized that HR-ASD infants would show overall larger P3 responses in the processing of the auditory stimuli, irrespective of stimulus type. In addition, we expected the enhancement of this component to be associated with later ASD-related traits. At the same time, given the similarities between the two disorders when considering both early language delay and their genetic etiology, we hypothesized finding common electrophysiological anomalies in the two groups of infants at high-risk (HR-ASD and HR-LI). We expected these common P3/MMR patterns to be related to the language outcome at later ages.

## Results

### Morphology of the STD, DEVF, DEVD and difference waveforms (MMRF and MMRD)

**Figure 1:**
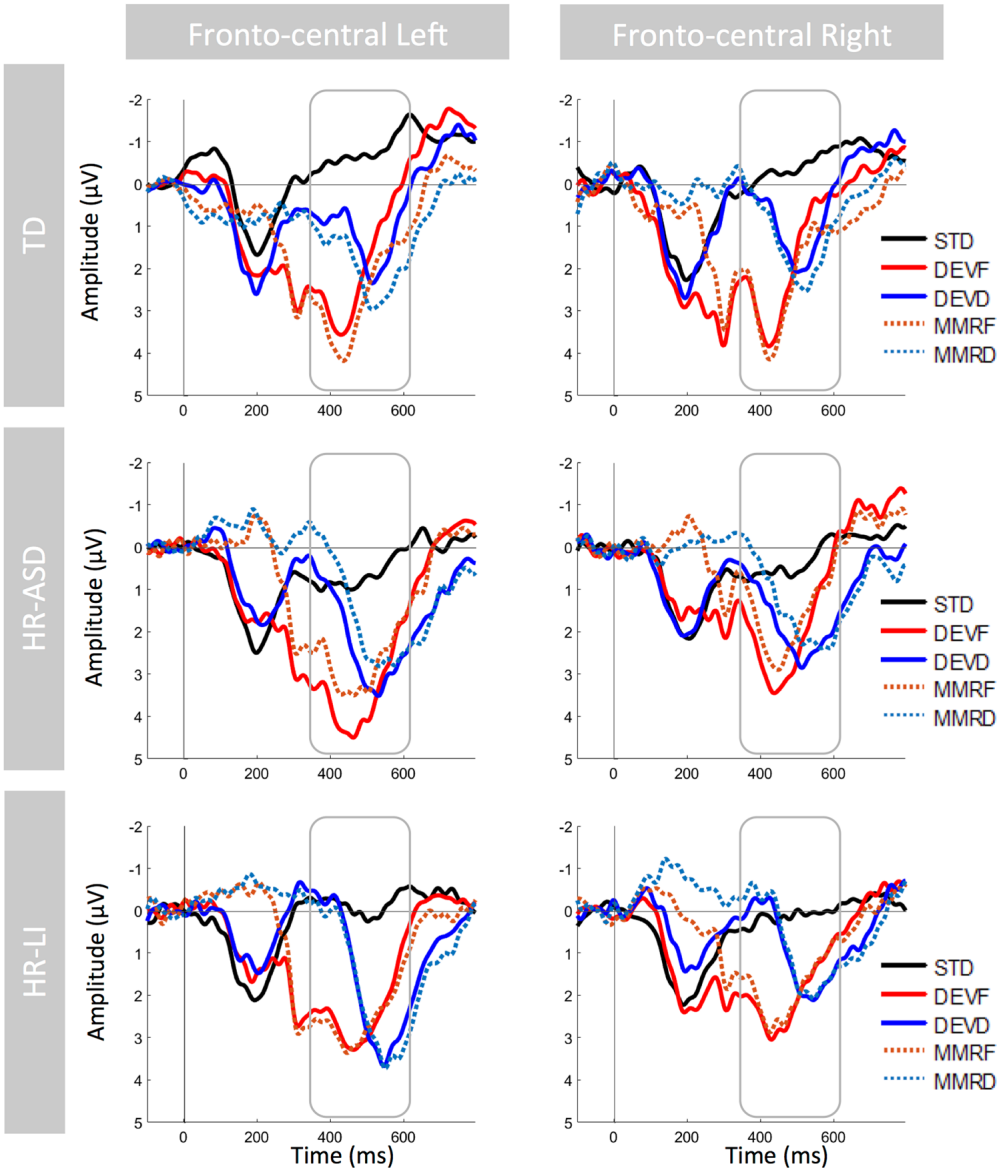
Grand average waveforms for the three groups. For graphic presentation purposes only, two virtual channels were created by merging ERPs from all the channels included in two clusters. The two virtual channels corresponding to the left and right fronto-central clusters are shown. The waveform relative to the standard condition (STD, black line) is plotted against the waveforms relative to the frequency deviant condition (DEVF, red line) and the duration deviant condition (DEVD, blue line). In addition, the difference waveforms relative to MMRF (DEVF minus STD; orange dotted line) and MMRD (DEVD minus STD; light-blue dotted line) are plotted. ERPs are time-locked to the onset of the first tone in the pair. Negative voltage is plotted upward. In order to highlight the significant results, the overall time-window of interest where mean amplitude was computed and peak latency was extracted (350–620 ms) is defined through squares.

**Figure 2:**
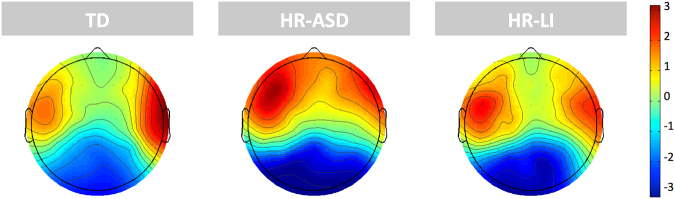
Topographical maps of the distribution of P3 amplitude for the three groups. Reflecting the statistical significant result (main effect of Group), the three Stimulus Types have been combined. Topographical distributions are shown for the Time-Window of interest for all the Stimulus Types (350–620 ms).

In all groups, there was a clear large positive peak (P3) occurring with different timing for each deviant stimulus type. Specifically, it appeared at approximately 450 ms for DEVF (and MMRF) and at about 520 ms for DEVD (and MMRD). In Fig. 1, grand average waveforms of the distribution of P3 amplitude and MMR latency in the two conditions (MMRF and MMRD) are shown. The topographical maps of the distribution of P3 amplitude are shown for the three groups in Fig. 2. Furthermore, descriptive statistics of the P3 amplitude and MMR latency separated by Group (i.e., TD, HR-ASD, HR-LI), hemisphere (i.e., Left, Right) and stimulus type (i.e., STD, DEVF, DEVD) are shown in Table 1.

**Table 1:** Descriptive statistics of the P3 amplitude and MMR latency separate for Group (HR-ASD, HR-LI, TD), Hemisphere (Left, Right) and Stimulus type (STD, DEVF, DEVD).

		TD n = 22 Mean (SD)	HR-ASD n = 20 Mean (SD)	HR-LI n = 19 Mean (SD)
P3 Mean Amplitude (µV)	STD Left	−0.56 (1.64)	0.84 (1.69)	−0.06 (2.11)
	STD Right	−0.22 (1.44)	0.59 (1.83)	0.18 (1.56)
	DEVF Left	2.50 (2.91)	3.83 (2.96)	2.75 (3.41)
	DEVF Right	2.40 (2.88)	2.55 (3.48)	2.32 (2.82)
	DEVD Left	1.31 (2.01)	2.73 (2.17)	2.33 (2.01)
	DEVD Right	1.17 (2.29)	2.19 (3.29)	1.32 (1.66)
MMR Peak Latency (ms)	MMRF Left	430.28 (32.50)	456.29 (38.46)	451.42 (43.44)
	MMRF Right	433.11 (30.69)	444.69 (30.54)	451.63 (36.67)
	MMRD Left	526.40 (49.12)	535.36 (32.20)	543.35 (26.09)
	MMRD Right	508.77 (32.89)	530.16 (32.04)	531.02 (34.23)

Note. MMR = mismatch response; MMRF = mismatch response frequency deviant; MMRD = mismatch response duration deviant; STD = standard stimuli; DEVF = deviant for frequency; DEVD = deviant for duration.

### Analyses of the ERP waveforms

#### Mean amplitude

When contrasting the P3 mean amplitude for the STD and DEVF stimuli by Hemisphere and by Group (2 × 2 × 3 ANOVA, Model 1), only a significant main effect of Stimulus type was found, *F*_(1,58)_ = 61.75, *p* < 0.001, ŋ^2^ = 0.516. As expected, mean amplitude was significantly higher for DEVF (M = 2.72, SD = 2.62) than STD (M = 0.11, SD = 1.55).

**Figure 3:**
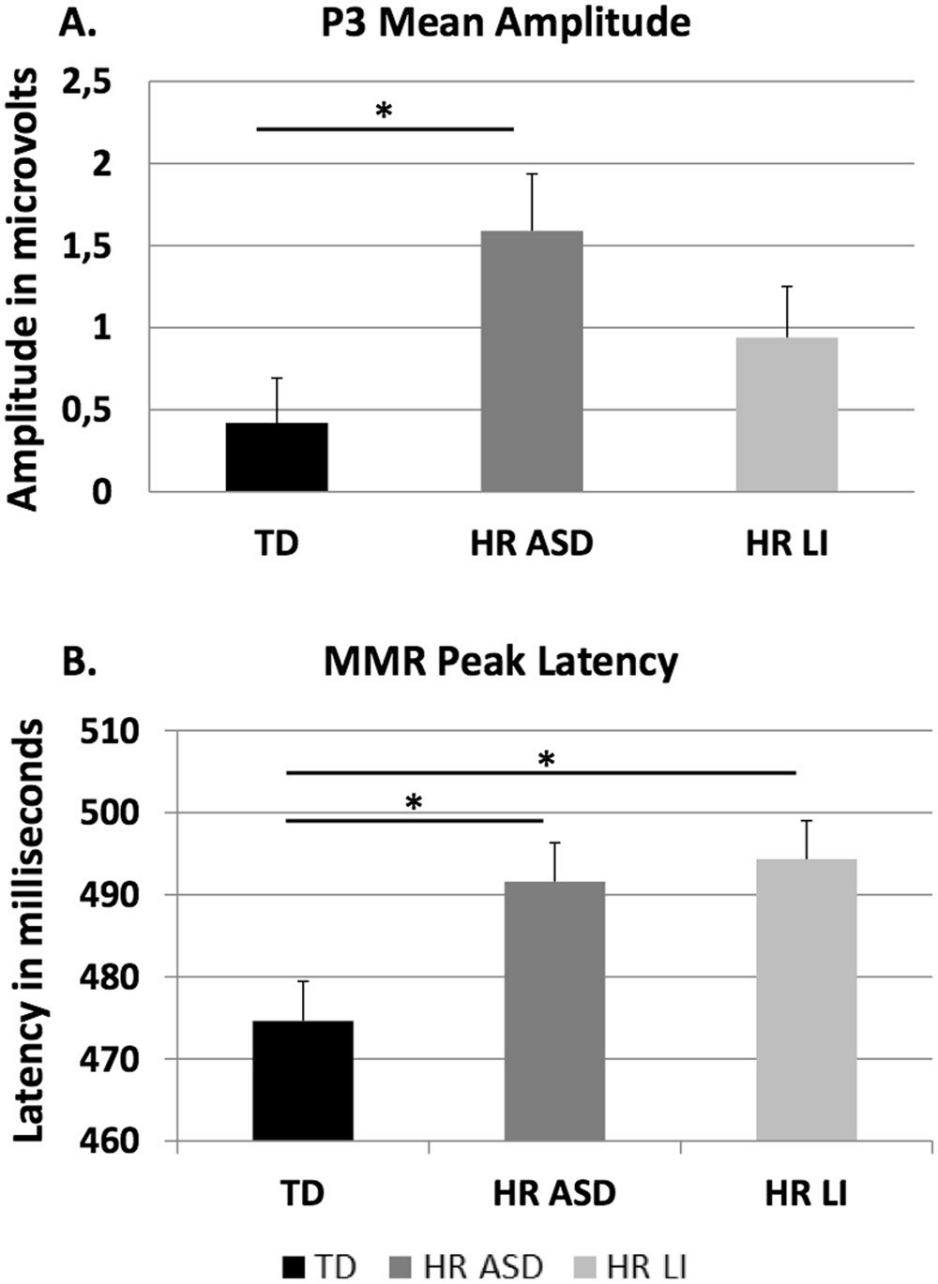
Bar graphs representing the significant main effect of Group for: (**A**) Mean amplitude (μV) of the P3 component (Model 2, contrasting STD and DEVD), and (**B**) Peak latency (ms) of the MMR. Error bars represent standard error of the mean. Significant differences between groups are reported (*<0.05, Bonferroni correction applied).

Similar -analyses were conducted to compare the P3 mean amplitude for the STD and DEVD stimuli by Hemisphere and by Group (2 × 2 × 3 ANOVA, Model 2), revealing significant main effects of Stimulus type, *F*_(1,58)_ = 45.80, *p* < 0.001, ŋ^2^ = 0.441 and Group, *F*_(2,58)_ = 3.65, *p* = 0.032, ŋ^2^ = 0.112. As expected, mean amplitude was overall higher for DEVD (M = 1.82; SD = 1.94) than STD. Interestingly, Bonferroni corrected post-hoc comparisons following the main effect of Group revealed that overall mean amplitude (irrespectively of Stimulus type and Hemisphere) was higher for HR-ASD (M = 1.59, SD = 1.55) than TD infants (M = 0.42, SD = 1.28; Bonferroni corrected post-hoc *p* = 0.027, Fig. 3, Panel A). No other comparison was significant (*p* > 0.10). Specifically, overall mean amplitude for HR-LI (M = 0.94; SD = 1.36) was situated in an intermediate position between HR-ASD and TD.

#### Peak latency

The overall two-way ANOVA model contrasting peak latency of the two difference waveforms (MMRs) by Stimulus type (MMRF vs. MMRD), Hemisphere (left vs. right) and Group (TD vs. HR-ASD vs. HR-LI) revealed significant main effect of Stimulus types, *F*_(1,58)_ = 269.72, *p* < 0.001, ŋ^2^ = 0.823. As expected, peak latency was longer for MMRD (M = 528.66, SD = 29.89) than MMRF (M = 444.03; SD = 30.64). Interestingly, the main effect of Group was also statistically significant, *F*_(1,58)_ = 3.58, *p* = 0.008, ŋ^2^ = 0.152. Bonferroni corrected post-hoc comparisons revealed that overall peak latency for both HR-ASD (M = 491.62; SD = 21.10) and HR-LI (M = 494.35; SD = 20.37) infants was longer than peak latency for TD (M = 474.64; SD = 22.70; Bonferroni corrected post-hoc *p* = 0.039 and *p* = 0.015, respectively, Fig. 3, Panel B).

### Associations between ERPs and ASD/LI outcome at 20 months of age

**Figure 4:**
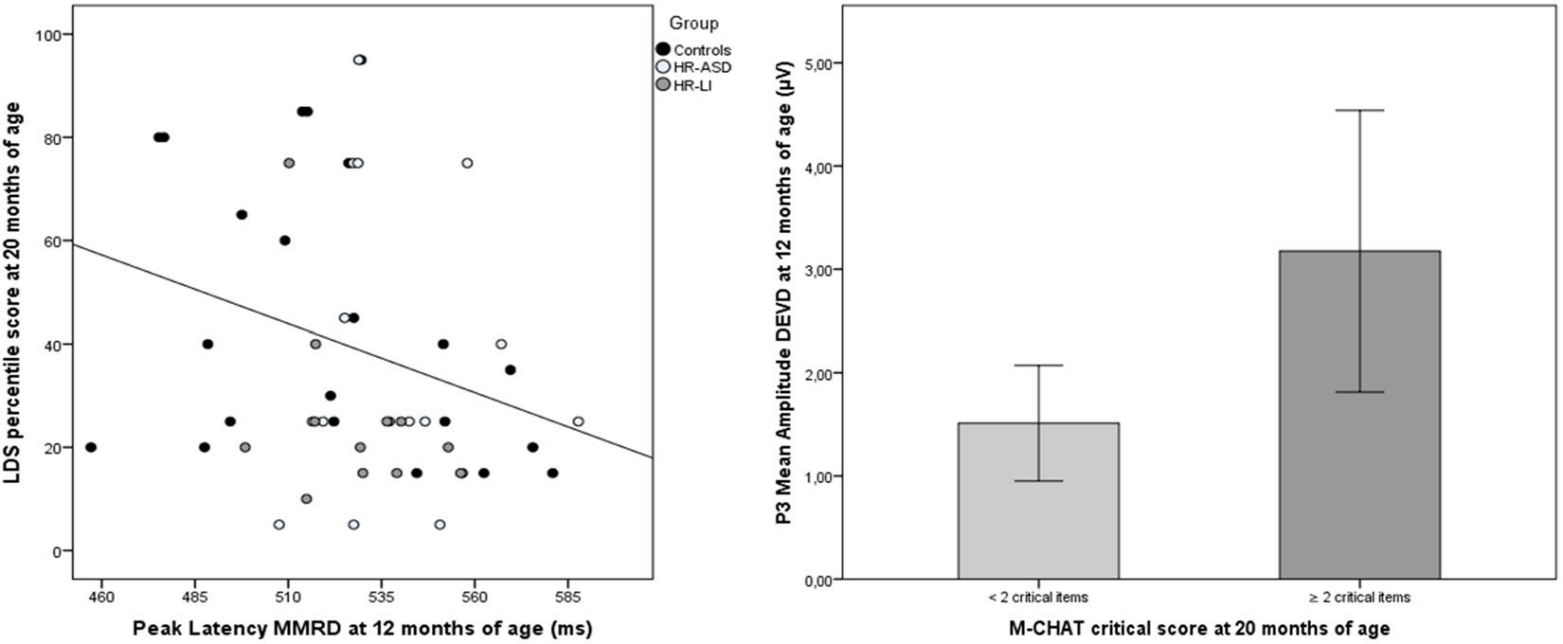
Association analyses between 12-month ERP components and 20-month LDS scores (Panel A) and M-CHAT critical scores (Panel B). Panel A represents Pearson’s correlation between MMRD latency and LDS percentile scores in all infants (black dots = controls; dark light-grey dots = HR-ASD group and dark-grey dots = HR-LI group). Panel B represents association between P3 DEVD mean amplitude in all infants and M-CHAT critical scores (coded as dichotomous variable, 0 = less than two M-CHAT critical items; 1 = equal or above two M-CHAT critical items).

The associations between ERPs components at 12-month-old and language/ASD-related outcomes at 20-month-old were assessed. Pearson’s correlations were performed between ERP measures at 12 months and LDS percentile score at 20 months, whereas non-parametric Mann-Whitney U test was used to examine the association between ERP measures at 12 months and M-CHAT at 20 months (coded as dichotomous variable, 0 = less than two M-CHAT critical items; 1 = equal or above two M-CHAT critical items^39^). For ERP measures, overall mean amplitudes of P3 for each stimulus type (i.e., STD, DEVF, DEVD) and overall peak latencies of MMR for each stimulus type (i.e., MMRF and MMRD) were entered in the association analyses. Follow-up data were available for 89% (*n* = 54), of the larger sample. Since there were no significant differences between groups on ASD/language outcomes (see Table 2), to maximize the statistical power the associations were computed using the combined groups. The analysis revealed that mean amplitude of P3 for DEVD condition was associated to M-CHAT critical scores, Mann-Whitney *U* = 99.00; *p* = 0.021 and peak latency of MMRD was associated to expressive vocabulary score, *r*_(54)_ = −0.291, *p* = 0.036. Infants with larger P3 amplitude at 12 months were characterized by higher scores in the M-CHAT screening test at 20 months (see Fig. 4A), whereas infants with faster MMRD at 12 months produced more words at 20 months (see Fig. 4B). No significant correlation was found between LDS and M-CHAT [*r*_(54)_ = 0.039; *p* = 0.783].

**Table 2:** Descriptive statistics and group comparisons on individual, demographic and clinical characteristics.

	TD (*n* = 22)	HR-ASD (*n* = 20)	HR-LI (*n = *19)	Group difference
Males/females	8/14	11/9	11/8	0.32^a^
Age (months)	12.40 (0.25)	12.36 (0.41)	12.50 (0.49)	0.48
Gestational age (weeks)	39.55 (1.65)	39.15 (1.09)	38.71 (1.61)	0.22
SES^b^	65.91 (13.42)	54.75 (16.18)	55.56 (25.20)	0.10
Expressive Language at 20 months^c^	44.00 (25.83)	44.38 (32.81)	23.93 (16.89)	0.06
M-CHAT critical items at 20 months	0.10 (0.45)	0.33 (0.82)	0.38 (0.77)	0.30^d^

Note. ^a^Chi-Square statistics; ^b^SES = socioeconomic status; SES was scored according to the Hollingshead 9-point scale, whereby a score ranging from 10–90 was assigned to each parental job and the higher of two scores was used when both parents were employed^69^; ^c^percentile scores in Language Development Survey (LDS^64^); ^d^Kruskal-Wallis test.

## Discussion

The present study aimed to directly compare two groups of 12-month-olds who were at high-risk for ASD and LI, at the electrophysiological level, and to associate their ERP responses with ASD-related traits and language outcome at 20 months. The results reported here are intriguing and unique, as this is the first study to provide abnormalities in ERP auditory processing profiles in ASD compared to LI high-risk infants and controls. In both ASD and LI at-risk groups, the latency of the MMR is delayed compared to controls. On the other hand, only the group at-risk for ASD shows overall larger P3 mean amplitude compared to controls. More interestingly, these ERP measures (regardless of risk status and clinical outcome) correlate with expressive vocabulary and critical M-CHAT items measured at 20 months of age in the whole sample.

A difference emerged when comparing infants with and without familial risk. Our results suggest that in both ASD and LI high-risk groups the peak latency of the MMR is delayed compared to controls, suggesting slower processing in both HR groups, specifically for fine-grained acoustic discrimination of changes in both frequency and duration. More interestingly, when we correlated the early ERP responses with later expressive language in the total sample, we found a significant association between the latency of the MMR for changes in duration and later expressive vocabulary.

In the domain of language and language-related impairments, our results are in line with previous studies that found that the ability to perform fine non-speech acoustic discrimination in early infancy is critically important to and highly predictive of later language development.

In both typically developing infants and in infants at familial risk for language/learning disorders (LLI)^29,30,32,33,37,40,41^, supporting auditory processing skills as early markers of risk and predictors of language development. Specifically, longer MMR latencies have been found in 2-month-old infants at risk for LLI in response to CV-syllables differing in vowel duration, suggesting delayed processing of the auditory stimulus change^40^. The latency of the mismatch negativity (MMN), which is often reported in older children and adults as an analog of the infants’ MMR^36^, has often been explored in older children with the full-blown diagnosis of LI^42^. Despite the methodological variation between studies leading to inconsistent results, at least two studies have reported markedly longer MMN latency in children with LI^43,44^.

On the other hand, the findings are still mixed in the ASD literature^45,46^, with some reports suggesting an intact mismatch response^47,48^ and others a delayed mismatch in ASD^45,46,49^, even if there are no infant studies, while convergent literature has suggested more prolonged mismatch latencies in ASD with concurrent LI^49,50^. Roberts and colleagues^49^ assessed MMR to tones and speech sounds in children with ASD and in children with ASD + LI. Interestingly, it was found that ASD children showed delayed latencies compared to controls, but a more pronounced delay was found in children with ASD + LI, supporting that the time course of the auditory mismatch may represent a biomarker for ASD, and specifically for language in ASD.

Taken together, our results seem to be in line with previous studies. Since the latency of MMR is similarly impaired in the two HR groups and is correlated with later language abilities, it is possible that delayed MMR indexes a dysfunction of auditory processing skills necessary for language development, thus representing a neural dysfunction common to language impairment in both ASD and LI.

In addition to the described electrophysiological pattern related to the latency of the MMR which seems to be common to the two at-risk groups, different ERP profiles for the two groups emerge concerning the amplitude of the P3 response. Only the group at-risk for ASD showed a larger P3 mean amplitude, primarily for STD and DEVD, compared to infants at-risk for LI and to controls. Furthermore, we found significant correlations between the P3 amplitude for duration changes and ASD-related outcome, suggesting the role of this ERP component as a specific predictor of later socio-communicative development.

These results appear to be consistent with prior evidence that reported a significant advantage in processing low-level auditory stimuli in ASD^51–53^. In particular, this result may be aligned with the “Enhanced Perceptual Functioning” theory^54^, positing that ASD is characterized by an enhanced perception of low-level perceptual information and that neural networks underpinning perceptual processing are “over-specialized”, which potentially results in a reduced tendency to integrate local information. One question arising from this result, though, is whether the findings are apparently in contrast with the idea that ASD is impaired in processing spectro-temporally complex auditory stimuli when high temporal demand is in place^24^, as is the case with our ‘deviant’ stimuli characterized by rapid changes^38^. It should be noted, however, that in our study the enhanced processing of the auditory information was not only found in response to ‘deviant’ stimuli, but additionally in response to the ‘standard’ stimuli. In the framework of the theoretical accounts of oddball processing^55^, the amplitude of the P3 component in response to ‘deviant’ stimuli is supposed to reflect the detection of the sensory stimulus feature mismatch and the following update of the stimulus representation. If no stimulus attribute change is detected (as in the case of STD stimuli), no update of the stimulus context is needed, and only sensory evoked potentials are recorded. For this reason, the larger amplitude found in our group at HR for ASD could be interpreted as increased sensory processing mechanisms, probably suggesting an overall enhanced sensitivity to low-level acoustic features, not specific for spectro-temporally complex changes.

Taken together, it could be that enhanced auditory processing might arise from a dysregulated sensory profile, supporting a possible hyper-sensitivity to perceptual level sensory features, especially for simple, low-level auditory stimuli. To further support our hypothesis, correlational analyses showed that different discrimination responses (larger P3 amplitude) are specifically associated with ASD-related phenotypes (i.e. M-CHAT). In particular, only the group at-risk for ASD showed overall larger P3 mean amplitude compared to controls and, regardless of risk status and clinical outcomes, a larger P3 amplitude was significantly associated with more ASD-related symptoms in all infants. Based on this result, we may hypothesize that enhanced neural responses to auditory discrimination processing very early in life carry significant and specific predictive value for ASD symptomatology during the course of development. Consistent with our results, a recent study^51^ demonstrated a relationship between enhanced discrimination of low-level perceptual contrasts and specific aspects of the ASD phenotype, measuring auditory discrimination ability in 29 adolescents with ASD, 26 with optimal outcomes (individuals diagnosed with ASD before age five, who had no symptoms at time of testing) and 20 controls. Interestingly, it was found that only the group with ASD showed enhanced pitch discrimination, compared with the other two groups, supporting the hypothesis that enhanced rather than impaired pitch discrimination may lead to ASD symptomatology. Intriguingly, in this study enhanced pitch discrimination also seemed to be associated with early language milestones (i.e. age at which children produced their first words), both in the group with ASD and in the optimal outcome group. Similar associations between atypically larger brain responses to speech and non-speech sounds and poor language scores were found in a recent MEG study conducted on school-aged children with ASD^53^. In addition, in a case-study, the brain responses to auditory stimuli were assessed in a non-verbal child with ASD, using both MEG and EEG^52^. The non-verbal child showed larger ERP responses to non-speech auditory stimuli, as compared to controls and to verbal children with ASD, and this result was interpreted as proof of the association between enhanced discrimination skills and lack of language. This association between enhanced discrimination skills and language impairment was not found in the present study, where distinct patterns of correlations were found between larger P3 amplitude and ASD-related traits, and between delayed MMR and language. Thus, our results are more aligned with the literature on individuals with or at-risk for language impairment^30^, suggesting language deficits to be associated with impaired, rather than enhanced, auditory processing skills. However, it is difficult to compare our results with previous literature, as there are no other ERP studies exploring the correlation between auditory processing and ASD-related traits and/or language skills in ASD at such an early age. Further studies are needed to confirm the present results and disentangle the pattern of associations.

Certain limitations should be taken into consideration. First, language outcomes and ASD related traits were assessed solely by parental report. Therefore, we suggest that future studies should employ direct assessment of language skills and autism-related symptoms. Second, the sample size in each group is relatively small and a replication in independent samples or using larger datasets may further refine the observed effects. In particular, since the incidence rate of ASD among infants identified as HR based on familial risk is about 20% for ASD^4,5^ and about 30% for LI^56^, it would be very interesting to examine developmental trajectories of P3/MMR components in a larger at-risk sample for ASD (who themselves will and will not ultimately be affected by ASD), as well as in a larger at-risk sample for LI (who themselves will and will not ultimately be affected by LI). Third, although we made an effort to match the three groups of infants for sex, it should be noted that the sample is not well representative of the male:female ratio commonly found in ASD and LI. Previous literature has shown that sex differences emerge in LI and ASD^4,57^, with boys more commonly diagnosed than girls, and in auditory processing^58^. Animal studies have shown that male mice with cortical malformations are impaired in auditory processing compared to controls, whereas females with the same induced malformations show no deficits^58^. It would be very interesting to test whether P3 responses significantly differentiate males from females. This remains an interesting hypothesis to be tested in future research with better suited sample sizes.

In conclusion, these results support the use of objective measurement of auditory processing to delineate specific pathophysiological mechanisms in ASD. They can help identify infants at-risk for ASD and possibly provide specific markers that help to distinguish ASD from linguistic delays. These results have strong implications for the future implementation of early intervention programs. A better understanding of shared and unique mechanisms underlying different disorders may support the development of more effective interventions by indicating whether interventions developed for one disorder are likely to be helpful for the another, and by identifying specific treatment targets that may be shared across disorders or are unique to each disorder.

## Methods

### Sample

At 12 months of age, 20 infants at high-risk for ASD (HR-ASD), 19 infants at high risk for LI (HR-LI) and 22 typically developing infants (TD) took part in the study. The TD group consisted of infants recruited by local advertisements in two hospitals in Northern Italy, whereas the HR groups were recruited at the Medea Institute. The recruitment of TD and HR-LI is part of a currently on-going longitudinal project aiming at identifying early risk markers for language and learning impairment^29,37,38^. Recruitment of HR-ASD was made possible through collaboration with the Italian Network for Early Detection of Autism Spectrum Disorders (NIDA Network).

All infants were included in the study if: (1) both parents were native-Italian speakers, (2) gestational age was ≥36 weeks, (3) birth weight was ≥2000 grams, (4) Bayley Cognitive Score^59^ was ≥7. The criterion for being included in the HR group was to have at least one sibling with a certified diagnosis of LI for the HR-LI group or a diagnosis of ASD for the HR-ASD group. There was no overlap in terms of recruited subjects.

Descriptive statistics of demographic and clinical characteristics are shown in Table 2. Participants were matched at the group level for sex, age, gestational weeks and socio-economic status (SES). Written informed consent was obtained from all parents prior to testing. The experiment was performed in accordance with relevant guidelines and regulations and was approved by the Medea Ethical and Scientific Committee.

### Follow-up assessment at 20 months old

Follow-up information on expressive language development and ASD-related traits was collected at about 20 months old (M = 20.75 months; SD = 0.69) by means of parental questionnaires.

### M-CHAT questionnaire

ASD-related traits were collected by M-CHAT questionnaire, a checklist to detect children at risk for ASD based on commonly observed child behaviors.

The M-CHAT is one of the most promising parental questionnaires commonly used in primary care and clinical settings^60–62^. It requires the parent to report on the presence of specific child behaviors in a checklist of 23 yes/no items. The presence of an abnormal behavior was assigned a score of 1 and the total score interpreted. Six items pertaining to social relatedness and communication (critical items) were found to have the best discriminability between children diagnosed with and without ASD. Thus, for the purpose of this study, children were considered as showing symptoms of ASD if 2 or more critical items were “no” (M-CHAT critical scoring^39^). The critical items are: (1) Does your child take an interest in other children? (2) Does your child ever use his/her index finger to point, to indicate interest in something? (3) Does your child ever bring objects over to you (parent) to show you something? (4) Does your child imitate you? (e.g. if you make a face, will your child imitate it?) (5) Does your child respond to his/her name when called? (6) If you point at a toy across the room, does your child look at it?

### Language Development Survey (LDS)

Infants’ expressive language was rated by the Language Development Survey (LDS), which was standardized for the Italian population^63^. LDS includes 310 words arranged into 14 semantic categories (e.g. food, animals, people, vehicles) and contains high frequency words (e.g. daddy), as well as less common words (e.g. supermarket). The number of total words that the child produces corresponds to the total vocabulary score. Norms are available from 18 to 35 months of age^64^. Percentile scores were entered in the analyses.

### Experiment design

**Figure 5:**
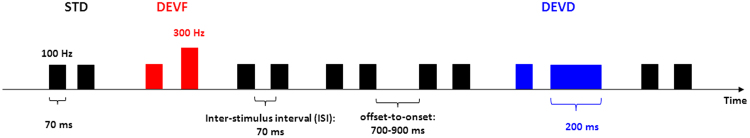
Schematic representation of the multi-feature oddball paradigm.

Auditory processing was assessed by means of an electrophysiological task tapping the ability to process rapidly changing and complex auditory stimuli. The paradigm and stimuli were identical to those used in previous research^29,37,38^. A non-speech multi-feature oddball paradigm was used in which pairs of brief complex tones with an inter-stimulus interval (ISI) of 70 ms were presented. Figure 5 schematically represents the experimental paradigm. The first tone in the pair always had a fundamental frequency of 100 Hz and duration of 70 ms. For ‘standard’ tone-pairs (STD stimuli) the same 100 Hz tone was repeated twice (i.e., 100–100 Hz). Two deviant tone-pairs differing with respect to the second tone were presented: in ‘deviant for frequency’ stimuli (DEVF), the second tone had a fundamental frequency of 300 Hz; in ‘deviant for duration’ stimuli (DEVD), the second tone had a duration of 200 ms. The stimuli were presented in a passive oddball paradigm in which 1200 stimuli (80% STD, 10% DEVF, 10% DEVD) were delivered in a pseudo-random order, so that at least three ‘standard’ tone-pairs were presented before each ‘deviant’ pair, with an inter-trial interval (offset-to-onset, ITI) varying randomly from 700 ms to 900 ms.

### EEG data acquisition and preprocessing

During EEG recording, the children were seated on their caregiver’s lap in a sound-attenuated and electrically shielded room. All stimuli were presented free field at an intensity of 75 dB via speakers located on either side of and equidistant (95 cm) from the subject. The ERP experiment lasted approximately 25 min., with an additional 10–15 min. before the experiment to prepare the participant for the EEG recording.

Auditory ERPs were recorded from 60 scalp sites using a dense-array EGI recording system (Electric Geodesic, Inc., Eugene, Oregon). Vertex was used as an online reference. EEG was sampled at 250 Hz and bandpass filtered (0.1–100 Hz) online. After recording, the data were processed using EEGLAB^65^ and ERPLAB^66^. The EEG data processing procedures were identical to those used in Cantiani and colleagues^29^. Continuous EEG data were bandpass filtered offline at 0.5–30 Hz. Channels with high impedance (>50 KΩ), or visually evident noise, were interpolated with a spherical spline (never more than 12 of the 60 channels). No interpolation was performed on the online reference Cz. The signals were then re-referenced to an average reference. For the STD, only responses to the immediate pre-deviant STD were included in the average. The continuous EEG was segmented according to stimulus type (pre-deviant STD, DEVF and DEVD) with 900 ms epoch lengths. The 100 ms pre-stimulus segment was used for baseline correction. Bad EEG epochs were identified and rejected using two automatic criteria, followed by visual inspection. First, a moving window (200 ms width, 50 ms step) was used to identify segments containing signals with voltage differences >150 μV. Then, trials whose spectrum (in one or more channels) deviated from the baseline by +25/−100 dB in the frequencies >20 Hz were removed^67^.

### Analytic strategy

To examine the role of auditory processing, we focused on the large positive response corresponding to the mismatch response (P3 component), reflecting a neural change detection process. Both amplitude and latency of this component were analyzed.

Time windows and electrode sites to be submitted to statistical analyses were selected based on mass univariate analyses applied to a subset of ERP data^29^. This procedure allows the identification of channel clusters and time windows where differences between stimulus types are significant, taking into account the application of appropriate corrections for multiple comparisons^68^ (for a full description of this procedure and the permutation test results that drove the selection of the time windows and the electrode sites to be submitted to statistical analyses, refer to Cantiani *et al*.^29^). For each participant, ERPs were extracted from a subset of 18 electrodes localized in the left and right fronto-central areas. Data were then averaged in two clusters corresponding to left and right fronto-central areas, each including nine channels (left cluster: E7, E9, E11, E12, E13, E14, E15, E18, E19; right cluster: E2, E3, E53, E54, E54, E57, E58, E59, E60; see Supplementary Information for the graphical representation of the selected electrodes and clusters placement map). Never more than 4 of the 18 channels included in the final fronto-central clusters involved in the analyses were interpolated (M = 0.6, SD = 0.9, range = 0–4). Following the above-mentioned mass-univariate analyses, mean amplitude was calculated for different time-windows: 350–550 ms for STD and DEVF and a 420–620 ms for DEVD. Mean amplitude was extracted within these time windows (instead of peak amplitude) because, as expected, clearly identifiable peaks were not present in the STD condition^29,38^. In addition, to investigate the latency of the component, peak latency was extracted in the same time-windows for the two difference waveforms (MMRF and MMRD). We decided to extract peak latency of the difference waveforms because, as previously stated, clearly identifiable peaks were not present in the STD condition.

### Statistical analysis

Differences in the mean amplitude of the P3 component were investigated using two separate 3-way ANOVA models in order to directly contrast each ‘deviant’ stimulus type with the ‘standard’^29^. This analytical strategy allowed us to investigate the amplitude of the mismatch response as a measure of auditory discrimination. Model 1 compared P3 mean amplitude to Stimulus Type (STD vs. DEVF) by Hemisphere (left vs. right) by Group (TD vs. HR-ASD vs. HR-LI; 2 × 2 × 3). Model 2 was a parallel analysis with STD and DEVD in Stimulus Type.

To explore latency differences, a 3-way ANOVA was again performed, directly contrasting peak latency of the two difference waveforms (MMRs). Specifically, an overall repeated-measures ANOVA model with Stimulus Type (MMRF vs. MMRD), Hemisphere (left vs. right) and Group (TD vs. HR-ASD vs. HR-LI) was used (2 × 2 × 3).

Finally, Pearson’s product moment correlation and Mann-Whitney U test analyses were conducted to assess associations between infant ERP components (mean amplitude of P3 and peak latency of MMR separated by stimulus type) (1) 20-month-old language abilities (expressive vocabulary percentile score - LDS) (2) 20-month-old screening score of ASD risk (M-CHAT).

## Electronic supplementary material

Supplementary File
