# Supplementary figures and images for: Distinct ERP profiles for auditory processing in infants at-risk for autism and language impairment
Source: Sci Rep. 2018 Jan 15;8:715. doi: 10.1038/s41598-017-19009-y (PMC5768787; doi:10.1038/s41598-017-19009-y)

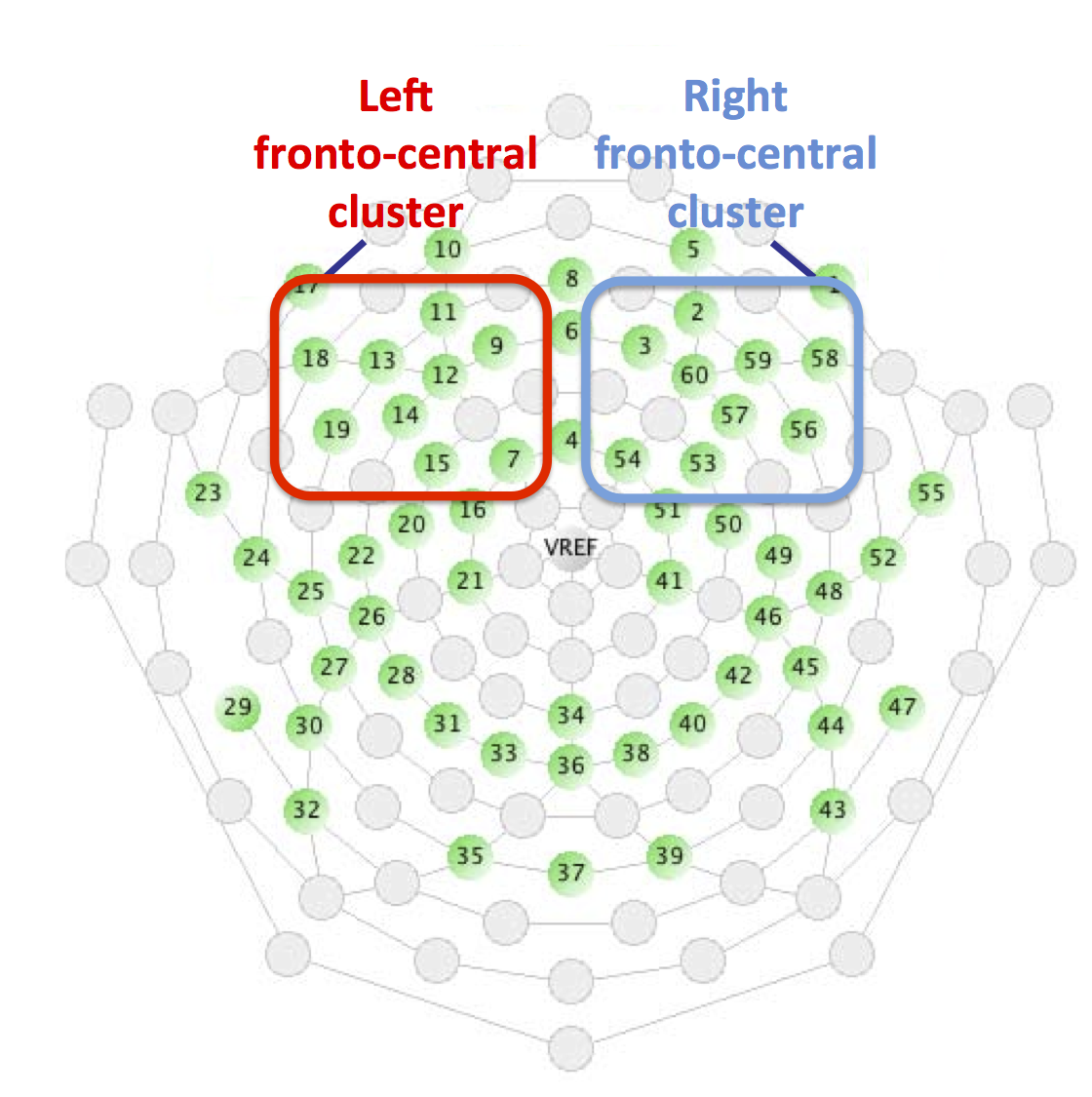

Supplement: Supplementary file 1 — Supplementary File [file 41598_2017_19009_MOESM1_ESM.tif]
